# Ormskirk Cottage Hospital

**Published:** 1897-02-13

**Authors:** 


### ORMSKIRK COTTAGE HOSPITAL.

MR. C. BEESTON, architect, writes: With reference to the criticism on the plan of this building appearing in your publication of January 23rd, I beg to call your attention to one or two points which, in justice to the charity as well as myself, should be corrected. (1) The wards at either end of the corridor are designed for three adults (not four), giving a cubic and floor space of 1,664 feet and 128 feet respectively. This is sufficient to allow for one child in addition in case of emergency. (2) An elementary principle of hospital construction was my guide, and I have availed myself of the opportunity and made provision for more than the recognised amount of light and air between each bed, although not shown on the published plan. In case of future extension this principle can be adhered to, whether the wards are extended east and west or towards the south. In the latter case the wards would take a double row of beds, with light and air between each. (3) The difficulty of the bath-room always exists in small cottage hospitals, which, as a rule, are not overburdened with subscriptions. Any difficulty with regard to the separation of the sexes can, I understand, be easily avoided by the nursing staff. (4) The doors opening into the vestibule, as well as all ward doors, are exactly the same width as the operating room, which is admitted to be a "reasonable width for its purpose." (5) Administration: The plan carried out was made in conformity with the instructions supplied to competitors. I also prepared a plan on similar lines showing the central block larger and two floors higher, with kitchen and other administration requirements, the two end wards being somewhat further apart.

*** Our architectural referee writes as follows in reply to the above: Mr. Preston has only himself to blame for the appearance of most of the criticisms to which he takes exception—some of which appear to have been fortunately met in the ultimate development of the building. With regard to his first point, a description forwarded by him (as "giving all particulars") with the plan for publication states that each of the wards has "space for four beds." No beds were shown on the plans, and the inference that they were intended for four beds each was obvious. We are glad they were not. As to his second point, we were, of course, guided by the plan sent us for publication. The windows there indicated are not beyond criticism, whether for an arrangement of three or four beds, whatever they may be in the building itself. His explanation of the third point does not remove the objection which we advanced to it, as a matter of arrangement. His fourth point is again due to the inaccuracy of the plan with which he favoured us, where the door into the vestibule from the corridor is shown considerably narrower than those of the wards or of the operating room. His fifth point merely leaves the existing plan fairly open to our criticism. In this he might, we think, as a successful competitor, have "bettered his instructions" in the event.

